# The modified shrinkage classification modes could help to guide breast conserving surgery after neoadjuvant therapy in breast cancer

**DOI:** 10.3389/fonc.2022.982011

**Published:** 2022-11-10

**Authors:** Zhao Bi, Peng-Fei Qiu, Tao Yang, Peng Chen, Xian-Rang Song, Tong Zhao, Zhao-Peng Zhang, Yong-Sheng Wang

**Affiliations:** ^1^ Shandong Cancer Hospital and Institute, Shandong First Medical University and Shandong Academy of Medical Sciences, Jinan, Shandong, People's Republic of China; ^2^ The First People’s Hospital of Lian Yun Gang, Radiotherapy Department, Xuzhou, Jiangsu, China

**Keywords:** breast cancer, neoadjuvant therapy, shrinkage, breast conserving surgery, prognosis

## Abstract

**Purpose:**

The traditional shrinkage classification modes might not suitable for guiding breast conserving surgery (BCS) after neoadjuvant therapy (NAT). Aim was to explore the modified shrinkage classification modes to guide BCS after NAT.

**Methods:**

From April 2010 to 2018, 104 patients were included. All patients underwent MRI examinations before and after NAT. Residual tumors were removed and divided into more than 30 tissue blocks at 5-mm intervals. After performing routine procedures for paraffin-embedded histology, we made semiserial sections (6-μm thick). The MRI and pathology 3D models were reconstructed with 3D-DOCTOR software. Combined with traditional shrinkage modes and efficacy of NAT, we derived modified shrinkage classification modes which oriented by BCS purpose: modified concentric shrinkage modes (MCSM) and modified non concentric shrinkage modes (MNCSM). The MCSM means the longest diameter of residual tumor was less than 50% and ≤2cm in comparison with the primary tumor before NAT. Other shrinkage modes were classified as MNCSM.

**Results:**

According to traditional shrinkage modes, 50 (48.1%) cases were suitable for BCS;while 70 (67.3%) cases were suitable for BCS according to the modified shrinkage modes (*p*=0.007). The consistency of MRI 3D reconstruction in assessing modified shrinkage classification modes was 93.2%, while it was 61.5% when assessing traditional shrinkage modes. Multivariate analysis showed that primary tumor stage, mammographic malignant calcification, molecular subtypes and nodal down-staging after NAT were independent predictors of modified shrinkage modes (all *p*<0.05). A nomogram was created based on these four predictors. With a median follow-up time of 77 months, the recurrence/metastasis rate in the MCSM and MNCSM group was 7.1% and 29.4%, respectively.

**Conclusion:**

Modified shrinkage classification modes could help to guide the individualized selection of BCS candidates and scope of resection after NAT. MRI 3D reconstruction after NAT could accurately predict modified shrinkage modes and extent of residual tumor.

## Introduction

Neoadjuvant therapy (NAT) is currently administered to patients with locally advanced breast cancers, to breast cancer of poor prognosis (triple-negative and HER2-positive tumors, or with nodal involvement and/or high proliferation rates), or to early-stage breast cancer having an indication of systemic therapy (1–4). A major clinical benefit of NAT is downstaging of the tumor. As a result, inoperable tumors may become operable and patients with large tumors could receive breast conserving surgery (BCS) to facilitate better cosmetic outcomes (2, 5, 6).

For patients who plan to receive BCS after NAT, the 5-year local-regional reference (LRR) rate was 2~7% in patients with tumor-free margins, but the risk increased to as high as 22% if the margin was positive (7, 8). Three strategies to mitigate the increased LRR after BCS in tumors downsized by NAT should be considered: careful tumor localization (including place marker clip, tumor range, and shrinkage modes), detailed pathological assessment, and appropriate radiotherapy (8). After NAT, tumor extent assessment can be difficult and shrinkage modes can be heterogeneous, making surgery technically more difficult than without NAT. So, for patients who plan to receive BCS after NAT, it is important to accurately assess residual tumor extent and shrinkage modes after NAT to ensure negative margins and reduce LRR as well as resection rate (9). The traditional view believed that patients with multinodular lesions, solitary lesion with adjacent spotty lesions and diffuse lesions were not suitable for BCS. In cases of multifocal residual tumor and/or cases of “scattered” residual tumor, the 2017 St. Gallen consensus conference expressed an opinion to favor more “generous” margins (10). However, the 2019 St. Gallen consensus conference recommended that the optimal resection remains removal of all known residual as opposed to original tumor lesions with a margin goal of “no ink on tumor” regardless of the presence of unifocal or multi-focal disease (11). That is to say, the traditional shrinkage classification modes would not sufficient as an indication for BCS.

Therefore, the aim of the present study is to explore and definite the modified shrinkage classification modes which oriented by BCS purpose after NAT.

## Patients and methods

### Patients

Between April 2010 to 2018, patients who treated at Shandong Cancer Hospital Breast Cancer Center were enrolled in this study. The study was approved by the Shandong Cancer Hospital Ethics Committee (No. SDTHEC20110324). Written informed consent was obtained from all patients before participation in the study, and all procedures were in accordance with the ethical standards of the responsible institutional committee on human experimentation and with the Helsinki Declaration. Adult women were included in this study if they 1) had histologically confirmed invasive breast carcinoma; 2) were clinical staging T_1-4_N_0-3_M_0_; 3) agreed to undergone NAT for the primary breast cancer. Patients were excluded according to the pre-established exclusion criteria if they had undergone therapy prior to NAT, concurrent cancer, bilateral breast cancer, or distant metastases.

**Figure 1:**
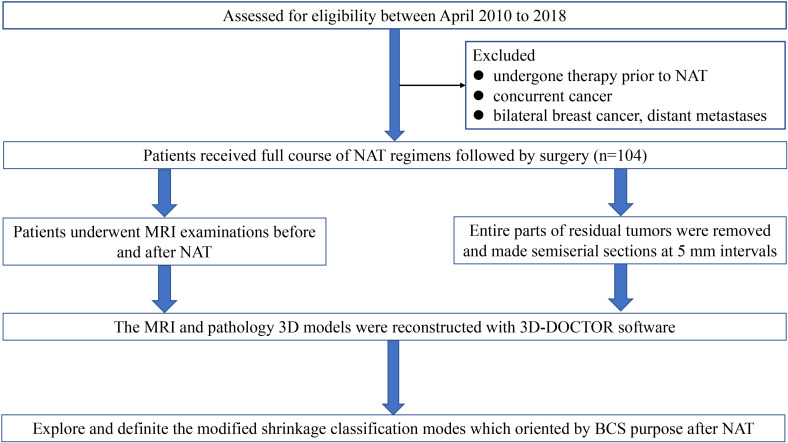
The consort diagram of the study.

In this study, we constructed the MRI and pathology three-dimensional (3D) reconstruction model of residual tumor. All patients underwent MRI examinations before and after NAT. The MRI and pathology 3D models of residual tumors were reconstructed with 3D-DOCTOR software. Then we explored and definite the modified shrinkage classification modes which oriented by BCS purpose after NAT, and assessed the advantage of modified shrinkage classification modes in guiding BCS after NAT. In addition, we assessed the accuracy of MRI 3D reconstruction in predicting the extent of residual tumor and modified shrinkage classification modes. Next, we analyzed the predictors of modified shrinkage classification modes, and generated a nomogram in predicting the modified shrinkage classification modes after NAT. The consort diagram of the study was illustrated in **Figure 1**.

### Treatment

Each patient underwent MRI examination twice, that is, before core biopsy and within 3 weeks after the last cycle of NAT. The mean interval time between the preoperative MRI examination and final surgery was 3 days (range 1~5 days).

Before NAT, all patients received core biopsy of the breast tumor and fine-needle aspiration of the clinical/image positive/suspicious axillary nodes guided by ultrasound. Hormone receptor (HR) was defined as positive with more than one percent expression rate. HER-2 receptor was considered as positive with immune-histochemical staining of 3+, or fluorescence *in situ* hybridization that was amplified (12). After these evaluations, molecular subtypes could be classified into Luminal A subtype, Luminal B HER2 negative (Luminal B HER2-) subtype, HER-2 positive (HER2+) and Tripe negative (TN) subtypes to precisely evaluate the biomarker effect.

All patients received standard dose four cycles of anthracycline and cyclophosphamide followed by four cycles of paclitaxel before surgery. HER2+ patients received anti-HER-2 targeted therapy.

### MRI acquisition and MRI 3D reconstruction

**Figure 2:**
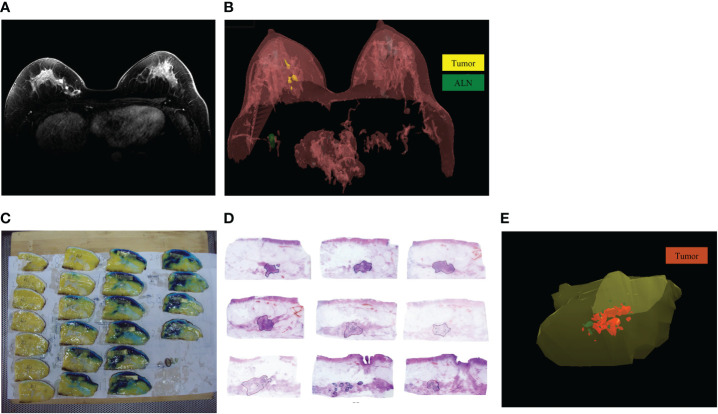
The MRI and pathology 3D reconstruction. **(A)**: The MRI imaging of residual tumor after NAT; **(B)**: The MRI 3D reconstruction model of residual tumor after NAT; **(C)**: The specimen was cut into several blocks at 5-mm intervals based on the markers; **(D)**: The extent of residual tumors was delineated under microscope; **(E)**: The pathology 3D reconstruction model of residual tumor after NAT.

MRI was performed using 3.0T scanners (Philips Medical Systems, Best, The Netherlands) with a dedicated 7 elements sense breast coil. Patients underwent imaging in the prone position with breast immobilized. Our imaging protocol included a localizing sequence followed by unilateral fast spin-echo T2-weighted imaging. The breast MRI imaging of all patients were independently assessed by one radiologist with 15 years of experience in reading breast MRI. He was unaware of the pathological outcomes and used the same measurement standard to measure the tumor size. In cases of rim enhancement, the necrotic core was included in measurement of the largest diameter. In cases of multifocal or diffuse tumor growth, the complete enhancing area, including intermediate (non-enhancing) tissue around the tumor, was measured on maximum intensity projection images. After scanning of the whole breast, bidimensional MRI images were transferred to 3D-DOCTOR software workstation to create and analysis 3D image of the breast (**Figure 2A**).

After delineating the extent of residual tumors in each MRI image, we chosen the command “3D Rendering/Surface Rendering/Simple surface”, then the MRI 3D model could be reconstructed (**Figure 2B**) (13). The shape and location of tumors in the breast and their relation to the adjacent tissues were examined. Using 3D images of MRI reconstruction model, the extent of each tumor was assessed by its largest diameter in three reformatted planes (sagittal, axial, and coronal) at initial and late enhancements. When there was no discernible contrast enhancement or a faint enhancement equal to the background normal tissue in previous tumor bed, this case was determined as radio complete response on MRI (14).

### Sub-serial sections of breast specimens and pathology 3D reconstruction

After mastectomy, according to the blue dye labeled extent of tumors before surgery, the tumor specimens were excised with a distance of 3.0 cm from the tumor boundary. After BCS, entire excised specimens were prepared for sub-serial sections.

The upper margins of specimens were marked with black ink, double-needle dyeing method to mark anchor points. Then the specimens were stored in a -20°C refrigerator. Then the specimen was cut into several blocks at 5-mm intervals based on the markers (**Figure 2C**). The tissue blocks were marked with continuous numbers and were immersed in 10% formalin solution for 48h. After performing routine procedures for dehydration and paraffin-embedded histology, we made one section of 4~6-μm thick in each block. The sections were cut using a Leica RM2010 slicer (Leica Biosystems, Nussloch, Germany) and stained with hematoxylin and eosin (13, 14).

Invasive tumors, calcification and ductal carcinoma *in situ* (DCIS) were delineated and recorded under microscope respectively (**Figure 2D**). The sections’ images were collected with the Epson V600 scanner (resolution 360 bpi) and stored as JPG format. The JPG data were integrated and calibrated based on anchor points using Photoshop software, then the sections’ images were imported into the 3D-DOCTOR software. With the “3D Rendering/Surface Rendering/Simple surface” command, the pathology 3D reconstruction model of residual tumor after NAT was presented (**Figure 2E**). The residual invasive tumor and calcification were marked with different colors in the pathology 3D reconstruction.

### The measurement of residual tumor

The longest diameter, maximum cross-sectional area and volume of residual tumors were measured according to the MRI and pathology 3D reconstruction models. The longest diameter refers to the longest distance in the 3D planes of residual tumors. Using the 3D-DOCTOR software, we select the “Boundaries in All Planes” command to project all the outlined tumor boundaries into the same plane. Then we measure the longest diameter and the longest vertical diameter of each plane. The maximum cross-sectional area would be calculated (the longest diameter × the longest vertical diameter). The volume could be automatically calculated by selecting the “Tools/Calculate Volumes” command from the 3D-DOCTOR software.

After surgery, the histopathological diagnosis of residual tumors was interpreted by two experienced pathologists. The tumor was evaluated using the Miller-Payne grading system. Surgical pCR was defined as no residual invasive tumor or DCIS within all slices (15).

### Shrinkage classification modes after NAT

**Figure 3:**
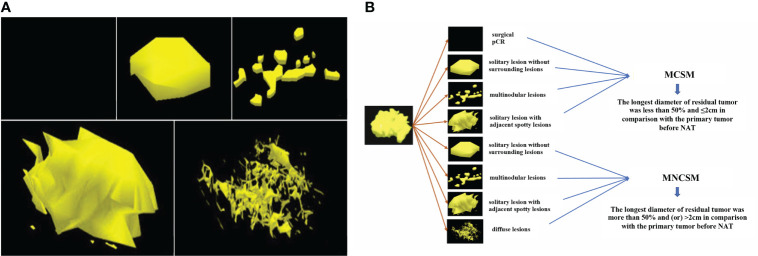
The shrinkage modes of residual tumors after NAT. **(A)**: The traditional shrinkage classification modes, including surgical pCR, solitary lesion without surrounding lesions, multinodular lesions, solitary lesion with adjacent spotty lesions and diffuse lesions. **(B)**: The modified shrinkage classification modes, including MCSM and MNCSM.

The traditional shrinkage classification modes of residual tumors after NAT were divided into five categories: surgical pCR, solitary lesion without surrounding lesions, multinodular lesions, solitary lesion with adjacent spotty lesions and diffuse lesions (**Figure 3A**) (16–19).

The BCS indications after NAT of MD Anderson Cancer Center (MDACC) include: ypT<2 cm, no vascular lymphatic invasion, single-focal lesions, and negative margins (20). Combined with the BCS indications of MDACC and traditional shrinkage classification modes, we derived and definite the modified shrinkage classification modes which oriented by BCS purpose: modified concentric shrinkage modes (MCSM) and modified non concentric shrinkage modes (MNCSM) (9). As the BCS indications after NAT of MDACC include diameter of residual tumor <2 cm, so we chose 2 cm as tumor size cutoffs. The MCSM means the longest diameter of residual tumor was less than 50% and ≤2cm in comparison with the primary tumor before NAT, including surgical pCR, solitary lesion without surrounding lesions, multinodular lesions and solitary lesion with adjacent spotty lesions. The longest diameter of residual tumor was more than 50% and (or) >2cm in comparison with the primary tumor before NAT were classified as MNCSM, including solitary lesion without surrounding lesions, multinodular lesions, solitary lesion with adjacent spotty lesions and diffuse lesions (**Figure 3B**). The modified shrinkage classification modes dismissed the shape, and focuses on efficacy and tumor extent, and our purpose was to guide BCS according to efficacy and tumor extent.

### Statistical analysis

For diagnostic accuracy based on the measurement of residual tumor size, the gold standard was defined as the pathology 3D reconstruction model-measured tumor size. Spearman rank correlation test and Bland-Altman method were used to evaluate the correlation and consistency between MRI and pathology 3D reconstruction measurement of residual tumor.

The association of different clinicopathological variables with modified shrinkage classification modes was analyzed. Pearson chi-square test or Fisher exact test was used to perform univariate analysis on categorical variables. Multivariable logistic regression analysis was conducted to identify the independent predictive factors of MCSM by using backward stepwise analysis.

A nomogram was developed based on variables in the final model with *p*<0.05 using “rms” package for R. Calibration of the nomogram was carried out by internal validation using the bootstrap resampling approach and was displayed using a calibration curve. The discrimination of the model was evaluated using the area under the curve (AUC) value of the ROC curve. Statistical analyses were performed using SPSS Statistics 22.0 software (IBM Corporation, Armonk, NY, USA) and R version 3.3.3 software (The R Foundation for Statistical Computing, Austria, Vienna). A *p*<0.05 was considered statistically significant.

## Results

### Patients’ characteristics

Between April 2010 to 2018, 104 patients received full course of NAT regimens followed by surgery in Breast Cancer Center. The median age of patients was 49 years old (rang 25 to 70 years). The clinical characteristics of the patients are summarized in **Table 1**.

**Table 1:** The clinical characteristics of 104 patients.

Characteristic		No.	%
Molecular subtypes			
	Luminal A subtype	23	22.1
	Luminal B HER-2 negative	21	20.2
	HER-2 positive	33	31.7
	Tripe negative	27	26.0
Clinical nodal stage			
	cN_0_	18	17.3
	cN_1_	41	39.4
	cN_2_	32	30.8
	cN_3_	13	12.5
Clinical tumor stage			
	cT_1_	11	10.6
	cT_2_	68	65.4
	cT_3_	13	12.5
	cT_4_	12	11.5
Breast surgery			
	Mastectomy	78	78.8
	BCS	22	21.2

### The different shrinkage classification modes after NAT

The traditional shrinkage classification modes presented by pathology 3D reconstruction were 34, 16, 19, 25, 10 cases among surgical pCR, solitary lesion without surrounding lesions, multinodular lesions, solitary lesion with adjacent spotty lesions and diffuse lesions, respectively (**Table 2**; **Figure 3A**). The modified shrinkage classification modes presented by pathology 3D reconstruction were 70 and 34 cases among MCSM and MNCSM, respectively.

**Table 2:** Patients according to the different shrinkage classification modes.

Shrinkage modes	MCSM (%)	MNCSM (%)	Total (%)
Surgical pCR	34 (48.6%)	0 (0)	34 (32.7%)
Solitary lesion without surrounding lesions	13 (18.6%)	3 (8.9%)	16 (15.4%)
Multinodular lesions	12 (17.1%)	7 (20.6%)	19 (18.3%)
Solitary lesion with adjacent spotty lesions	11 (15.7%)	14 (41.1%)	25 (24.0%)
Diffuse lesions	0 (0)	10 (29.4%)	10 (9.6%)
Total	70 (100%)	34 (100%)	104 (100%)

MCSM, modified concentric shrinkage modes; MNCSM, modified non concentric shrinkage modes

### The modified shrinkage classification modes were more suitable for guiding BCS

According to the traditional shrinkage classification modes, 50 (48.1%) cases in this study were suitable for BCS;while 70 (67.3%) cases were suitable for BCS according to the modified shrinkage classification modes (*p*=0.007, **Table 3**). According to the traditional shrinkage classification modes, patients with multinodular lesions and solitary lesions with adjacent spotty lesions were not suitable for BCS; while there were 52.3% (23/44) of them present with MCSM, they were still suitable for BCS. Among patients with solitary lesions without surrounding lesions, there were 18.8% (3/16) of patients present with MNCSM, they were still not suitable for BCS (**Table 2**).

**Table 3:** Candidates of BCS according to the different shrinkage classification modes.

Different shrinkage modes	Suitable for BCS (%)	Not suitable for BCS (%)	Total
Traditional shrinkage modes	50 (48.1%)	54 (51.9%)	104
Modified shrinkage modes	70 (67.3%)	34 (32.7%)	104

### MRI 3D reconstruction could accurately assess modified shrinkage classification modes

The accuracy, sensitivity, specificity, positive predictive value and negative predictive value of MRI 3D reconstruction in assessing traditional shrinkage modes were 84.6%, 61.9%, 90.4%, 61.9% and 90.4%, respectively (the consistency rate was 61.5%) (**Table 4**).

**Table 4:** The traditional shrinkage modes after NAT between MRI and pathology 3D reconstruction.

Traditional shrinkage modes	MRI 3D reconstruction	Pathology 3D reconstruction	
		+	-
Surgical pCR	+ -	26 (25.0%) 8 (7.7%)	7 (6.7%) 63 (60.6%)
Solitary lesion without surrounding lesions	+ -	8 (7.7%) 8 (7.7%)	19 (18.3%) 69 (66.3%)
Multinodular lesions	+ -	7 (6.7%) 12 (11.5%)	6 (5.8%) 79 (76.0%)
Solitary lesion with adjacent spotty lesions	+ -	18 (17.3%) 7 (6.7%)	8 (7.7%) 71 (68.3%)
Diffuse lesions	+ -	5 (4.8%) 5 (4.8%)	0 (0) 94 (90.4%)

The MRI and pathology 3D reconstruction had a high consistency in assessing modified shrinkage classification modes (the consistency rate was 93.2%). The accuracy, sensitivity, specificity, positive predictive value and negative predictive value of MRI 3D reconstruction in assessing the modified shrinkage classification modes were 93.3%, 97.0%, 86.5%, 92.9% and 94.1%, respectively (**Table 5**).

**Table 5:** The modified shrinkage classification modes after NAT between MRI and pathology 3D reconstruction.

Pathology 3D reconstruction	MRI 3D reconstruction		Total
	MCSM	MNCSM	
MCSM	65 (62.5%)	5 (4.8%)	70 (67.3%)
MNCSM	2 (1.9%)	32 (30.8%)	34 (32.7%)
Total	67 (64.4%)	37 (35.6%)	104 (100%)

### MRI 3D reconstruction could accurately assess residual tumor extent

For diagnostic accuracy based on the measurement of residual tumor size, the gold standard was defined as the pathology 3D reconstruction model-measured tumor size. The correlation among the longest diameter, maximum cross-sectional area and volume of residual tumors after NAT measured by MRI and pathology 3D reconstruction has statistically significance, respectively. And the *r* value was 0.942, 0.941 and 0.903, respectively (all *p*<0.001). In terms of the largest diameter and largest cross-sectional area of the residual tumor, the correlation between 3D pathology and 3D MRI was better than two-dimensional MRI measurement (**Table 6**).

**Table 6:** Different examination methods to measure the largest diameter and cross-sectional area of residual tumor after NAT [M (95%CI)].

Methods	Largest diameter (cm)	Largest cross-sectional area (cm^2^)
2D MRI	1.25 (0.785-1.955)	1.107 (0.655-1.817)
MRI 3D reconstruction	1.40 (0.810-2.010)	1.250 (0.750-1.850)
Pathology 3D reconstruction	1.45 (0.812-2.050)	1.312 (0.801-1.930)

Compared with pathology 3D reconstruction, MRI 3D reconstruction slightly underestimated the maximum diameter and maximum cross-sectional area of residual tumors, with a median disparity (*MD*) of -0.074cm (95% CI: -0.313~0.165cm) and -1.148cm^2^ (95% CI: -2.146~ -0.148 cm^2^). And it overestimated the volume of residual tumors compared with pathology 3D reconstruction, with *MD* of 0.433 cm^3^ (95%CI: -9.55~12.34 cm^3^).

### The nomogram of modified shrinkage classification modes after NAT

The authors generated a unique, random number using a computer for each patient included. And authors sorted patients according to their random numbers. Finally, 71 patients with smaller numbers were assigned to the training set, and the other 33 patients were assigned to the validation set.

In the training set, although there was no important difference in year and menopausal status among the modified shrinkage classification modes after NAT, significant difference between modified shrinkage classification modes and primary tumor stage before NAT (*p*=0.009), clinical nodal stage after NAT (*p*=0.013), lymph nodes downstaging after NAT (*p*<0.001), mammographic malignant calcification (*p*=0.002) and molecular subtypes (*p*<0.001) were observed in univariate analysis. Variables with *p*-value<0.05 in the univariate analysis were assessed for multivariate analysis. The independent predictors of modified shrinkage classification modes were comprised of primary tumor stage (OR=2.059, 95%CI: 1.187-3.574, *p*=0.001), mammographic malignant calcification (OR=3.424, 95%CI: 1.437-8.161, *p*=0.005), molecular subtypes (OR=0.530, 95%CI: 0.364-0.772, *p*=0.001) and nodal down staging after NAT (OR=0.183, 95%CI: 0.067-0.497, *p*=0.010) (**Table 7**).

**Table 7:** The predictive factors for modified shrinkage classification modes after NAT in the training cohorts.

Factors	MCSM	MNCSM	Univariable analysis	Multivariable analysis
			*p* value	*p* value
Clinical tumor stage			0.009	0.001
cT1	7	1		
cT2	33	14		
cT3	4	4		
cT4	3	5		
Clinical nodal stage			0.659	
cN0	8	4		
cN1	17	11		
cN2	16	6		
cN3	7	2		
Nodal stage after NAT			0.013	
ycN0	27	7		
ycN1	12	5		
ycN2	5	5		
ycN3	5	5		
Molecular subtypes			0.001	0.001
Luminal A	5	10		
Luminal B HER2-	9	5		
HER2+	18	5		
TN	16	3		
Lymph nodes downstaging			0.001	0.010
Yes	43	14		
No	6	8		
Malignant calcification			0.002	0.005
Yes	20	16		
No	29	6		

**Figure 4:**
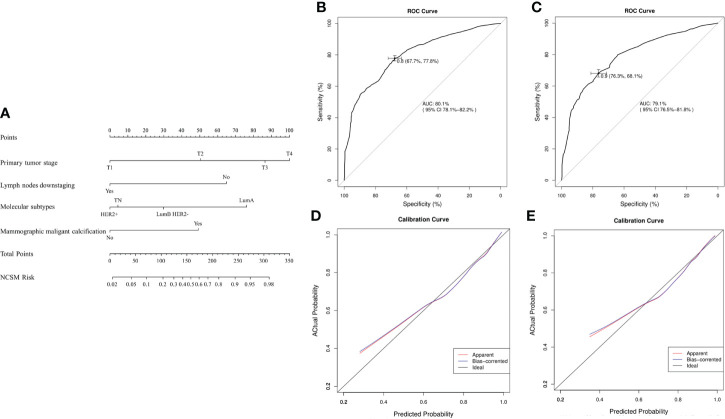
The nomogram to predict modified shrinkage classification modes after NAT. **(A)**: To calculate the probability of MNCSM, the scores for the four factors were summed up. And the total scores and bottom risk scale were referenced. **(B)**: The ROC curve in the training cohort indicates an AUC of 0.801. **(C)**: In the validation cohort, the ROC curve indicates an AUC of 0.791. The calibration curve showed a satisfactory fit between the actual and predicted probability of achieving MNCSM in the training **(D)** and validation **(E)** cohorts. The horizontal axis indicates the predicted probability measured by the nomogram, and the vertical axis indicates the actual probability.

Based on the aforementioned multivariate analysis results, the authors built the nomogram to predict patients with MNCSM (**Figure 4**). To calculate the probability of MNCSM, the scores for the four factors were summed up. And the total scores and bottom risk scale were referenced (**Figure 4A**). The overall performance and discriminative performance of the model were assessed by the calibration curve and ROC curve analysis, respectively. The nomogram was internally validated using the bootstrap method. The nomogram had an AUC of 0.801 (95% CI: 0.781–0.822) in the training set, indicating that the multivariate logistic regression model had potentially promising predictive power (**Figure 4B**).

The external validation set of 33 patients also showed good discriminatory ability, with an AUC of 0.791 (95% CI: 0.765–0.818), indicating that use of a multivariate logistic regression model in an individual set had potentially promising predictive power (**Figure 4C**). The difference between the two AUCs was not statistically significant (*p*=0.778). The calibration curve showed a satisfactory fit between the actual and predicted probability of achieving MNCSM in the training (**Figure 4D**) and validation (**Figure 4E**) sets, indicating that the nomogram was well calibrated.

### Patients with MNCSM had higher recurrence/metastasis

The median follow-up was 77 months (40-134 months), with the last follow-up in May 2022. Twelve cases were lost to follow-up, and the effective follow-up rate was 88.5% (92/104). We observed 5 cases of recurrences/metastasis (7.1%) in the MCSM group and 10 cases (29.4%) in the MNCSM group (*p*=0.002). In MCSM group, one patient had chest wall recurrence and 4 patients had distant metastases. While in MNCSM group, 2 patients had chest wall recurrence, and 8 patients had distant metastasis. At the same time, the multivariate analysis also showed that modified shrinkage modes were the independent predictors of recurrence/metastasis.

### Patients with MCSM had better survival benefit

**Figure 5:**
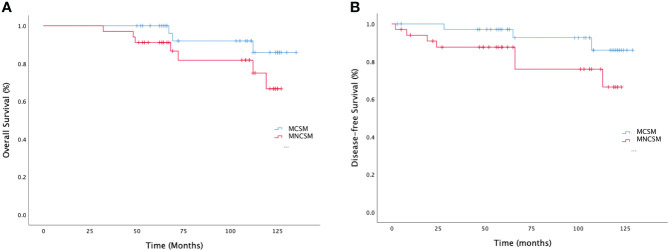
The survival analysis of modified shrinkage classification modes. **(A)**: The overall survival of MCSM and MNCSM among patients without pCR. **(B)**: The disease-free survival of MCSM and MNCSM among patients without pCR.

Previous studies had confirmed that pCR after NAT was associated with survival benefits. So, we want to exclude the effect of pCR on the survival benefit of MCSM group. We performed a subgroup analysis to assess survival benefit of patients who did not achieve pCR. The median overall survival (OS) in MCSM and MNCSM group was 108.5 months and 89.0 months, respectively (**Figure 5A**). The median disease-free survival (DFS) was 101.5 months and 60.5 months, respectively (**Figure 5B**) in patients with MCSM and MNCSM. Even patients with MCSM did not achieve pCR, they also had a better survival benefit compared to patients with MNCSM.

## Discussion

The continuous optimization of local-regional control under the guidance of molecular subtype allows clinicians to make reasonable adjustments based on the efficacy of NAT to achieve the maximum treatment benefits. In this study, we constructed the MRI and pathology 3D reconstruction model of residual tumor after NAT. Then we explored and definite the modified shrinkage classification modes which oriented by BCS purpose after NAT, we found that modified shrinkage classification modes were more suitable for guiding BCS after NAT. At the same time, based on the gold standard of 3D pathology reconstruction model-measured tumor size, we also found that MRI 3D reconstruction after NAT could accurately predict the modified shrinkage classification modes and extent of residual tumor. In addition, a nomogram was developed based on the predictors of modified shrinkage classification modes that might aid clinicians in surgical decisions. The nomogram indicated that patients with large primary tumor, mammographic malignant calcification, Luminal A/Luminal B HER2- subtype, and high nodal burden after NAT were more likely to present with MNCSM. With an AUC of 0.801 and internal validation using the bootstrap resampling method, the model exhibited sufficient ability to predict modified shrinkage modes after NAT. Patients with MCSM had better survival benefit.

The main strength of the study was that we constructed the BCS-oriented modified shrinkage classification modes which combined traditional shrinkage modes with residual tumor extent. Compared with traditional shrinkage modes, modified shrinkage classification modes were more suitable to guide the individualized selection of BCS candidates and scope of resection. This mode could help to decrease the negative margins distance and simultaneously maintain the natural breast shape to facilitate better cosmetic outcomes. And it represents a transformation of treatment concept, which from maximum and tolerable treatment to the minimum and effective treatment (11).

The single-focal lesion was one of the BCS indications after NAT of MDACC (20). The traditional view believed that multinodular lesions and solitary lesion with adjacent spotty lesions were not suitable for BCS. However, in our study, for patients with a high probability of MCSM after NAT, even if they had multinodular lesion or solitary lesion with adjacent spotty lesion, BCS would also be safe if they had a negative margin. So, our study might partly expand the indications of BCS after NAT: patients might also accept BCS safely even if they had multi-focal disease after NAT. And for these patients, there would be no increase in LRR if they received BCS successfully. For patients with a high probability of MNCSM, the basic goal of NAT (tumor downstage) had not been achieved. If satellite lesions were missed during surgery, LRR would increase due to “false negative margins”. So, these patients need to be cautious when choosing BCS, at the same time, they also need a more “generous” resection extent. The 2019 St. Gallen consensus conference also recommended that patients with multi-focal disease could also accept BCS after NAT, but the scope of residual tumors need to be more accurately assessed.

The MRI 3D reconstruction model provides an intuitive image of tumor extent in the breast and is helpful for surgeons to plan surgery. Furthermore, it can display more precise information than routine bidimensional images, because 3D tumor images can be observed from various directions by rotation (20–24). Taking advantage of these characteristics, MRI 3D reconstruction has a high degree of accuracy in assessing the residual tumor extent after NAT. Several reports have demonstrated that MRI 3D reconstruction significantly and strongly correlated with pathology examination (25, 26). Chae YL et al. (25) evaluated the accuracy of 3D measurement by computer-aided program of breast MRI for the assessment of residual tumor extent. The result showed that there was no significant difference between the 3D measurement and histological diameter. Kenji et al. (26) also found that the tumor size determined by 3D MRI showed a strong correlation with that determined by pathologic examination (r=0.896). However, most of them compared the tumor extent which was assessed by its largest diameter at MRI 3D reconstruction model with the pathology examination of routine sliced images. The pathology 3D reconstruction has also been previously used in researches, and it could also provide more precise information about tumor extent than routine sliced images (13, 14, 27, 28). However, as far as we know, few studies compared the association and correlation between MRI 3D reconstruction and pathology 3D reconstruction in evaluation of residual tumor extent. In this study, taking pathology 3D reconstruction-measured tumor size as the gold standard, we further confirmed the accuracy of MRI 3D reconstruction in assessing residual tumor extent after NAT. At the same time, the MRI images were easy to obtain, and the 3D reconstruction technology was relatively mature. So, we recommend applying MRI 3D reconstruction techniques to evaluate residual tumor extent after NAT in clinical practice.

Our results also showed that molecular subtype was an independent predictor of the modified shrinkage classification modes. Patients with Luminal A and Luminal B HER2- subtypes had more chance to achieve MCSM. The correlation between molecular subtype and modified shrinkage modes might reflect tumor biologic characteristics. One possible reason might be the growth characteristic of Luminal A and Luminal B HER2- subtypes, tumor cells tend to grow slowly with low apoptosis rate and genetic instability (17). Simultaneously, tumor cells in these subtypes may be more resistant to preoperative therapy. However, tumor cells in TN and HER2+ subtypes had poor differentiation and strong proliferation ability, the aggressive tumor cells were more sensitive to therapy (29). After NAT, the tumor boundary of patients with MCSM was easy to judge, and the margins of these tumors were often negative after finishing tumor resection. But the tumor boundary of patients with MNCSM is difficult to determine accurately. For those patients, LRR might be increased due to “false negative margin” when performing BCS. Therefore, Luminal A and Luminal B HER2- patients with large primary tumor and/or high nodal burden after NAT should be cautious to receive BCS after NAT, and the negative margin distance might also need to be appropriately increased. Although some patients with TN and HER2+ subtypes had the poor prognosis, patients with these subtypes were more likely to present with MCSM after NAT, suggesting that BCS after NAT was also feasible for TN and HER2+ patients.

Shrinkage classification modes were reported to be associated with prognosis. Ippei et al. (17) found patients with concentric shrinkage pattern has an excellent DFS (*p*=0.007) and OS (*p*=0.037). Our study also found that the modified shrinkage classification modes might be related to the prognosis. Patients with MNCSM might have a worse prognosis. The reasons might be that the predictors associated with MNCSM indicated high tumor burden, and these predictors were associated with poor prognosis. Another reason might be that molecular subtype was associated with DFS and OS after NAT. The result of Orsaria et al. (30) showed that patients with pCR after NAT had better DFS, particularly for HER2+ and TN subtypes. Zarotti et al. (31) also found that clinicopathological factors and distinct therapy regiments especially in HER2+ and TN subtypes had prognostic impact on pCR, OS and DFS after NAT. In our study, patients with HER2+ and TN subtypes had more chance to achieve MCSM, these patients also had a better DFS and OS after NAT.

This study has certain limitations, and the most important of which is the small sample size. Additionally, lacking multi-center external data to verify the accuracy of the nomogram is another limitation in our study. Therefore, further prospective multi-center studies are required to confirm and assess the results of modified shrinkage classification modes.

## Conclusion

The modified shrinkage classification modes could help to guide the individualized selection of BCS candidates and scope of resection after NAT. MRI 3D reconstruction after NAT could accurately predict the modified shrinkage classification modes and extent of residual tumor. The nomogram combined clinical factors, imaging, molecular subtypes and NAT efficacy showed sufficient predicting accuracy in predicting modified shrinkage classification modes. Patients with MNCSM after NAT might have a worse prognosis.

## Data availability statement

The datasets presented in this study can be found in online repositories. The names of the repository/repositories and accession number(s) can be found in the article/supplementary material.

## Ethics statement

Written informed consent was obtained from the minor(s)’ legal guardian/next of kin for the publication of any potentially identifiable images or data included in this article.

## Author contributions

All authors contributed to the study conception and design. Material preparation, data collection and analysis was performed by BZ, QPF, YT, and CP. The first draft of the manuscript was written by BZ and ZZP, and all authors commented on previous versions of the manuscript. All authors contributed to the article and approved the submitted version.

## Funding

This work was funded by National Natural Science Foundation of China (82172873, 81672104), Shandong Cancer Hospital and Institute Clinical Training Program (2020PYA08), National Natural Science Foundation of Shandong Province (ZR2021QH002), China Postdoctoral Science Foundation (2021M691334), Bethune Foundation Project (G-X-2019-0101-12), Shandong Province Medical and Health Science and Technology Development Plan Fund (2019WS201).

## Conflict of interest

The authors declare that the research was conducted in the absence of any commercial or financial relationships that could be construed as a potential conflict of interest.

## Publisher’s note

All claims expressed in this article are solely those of the authors and do not necessarily represent those of their affiliated organizations, or those of the publisher, the editors and the reviewers. Any product that may be evaluated in this article, or claim that may be made by its manufacturer, is not guaranteed or endorsed by the publisher.
